# Butyrate-Producing Bacteria as a Keystone Species of the Gut Microbiome: A Systemic Review of Dietary Impact on Gut–Brain and Host Health

**DOI:** 10.3390/ijms27031289

**Published:** 2026-01-28

**Authors:** Jacob L. Snodgrass, Bisi T. Velayudhan

**Affiliations:** Department of Biology, James Madison University, Harrisonburg, VA 22807, USA

**Keywords:** butyrate-producing bacteria, gut–brain axis, gut health

## Abstract

The human gut microbiome is a complex ecosystem integral to host health, with butyrate-producing bacteria (BPB) playing a critical role in maintaining intestinal homeostasis. This scoping review explores the composition, function, and systemic influence of BPB, focusing on their metabolic product, butyrate, and its implications for gut integrity, immune modulation, and gut–brain axis (GBA) communication. Disruptions to BPB abundance, which is correlated with Western dietary patterns, food additives, and antibiotic exposure, are linked to gut dysbiosis and associated with a wide spectrum of chronic diseases, including inflammatory bowel disease (IBD), obesity, type 2 diabetes, neurodegenerative disorders, and psychiatric conditions. Butyrate supports colonocyte energy metabolism, reinforces epithelial barrier function, regulates goblet cell mucus production, and exerts anti-inflammatory effects via histone deacetylase inhibition and G-protein-coupled receptor signaling. The depletion of BPB and the resultant butyrate deficiency may represent a unifying pathophysiological mechanism underlying these conditions. Therapeutic strategies that restore BPB populations and butyrate levels, such as prebiotics, dietary fiber, and microbiota-targeted interventions, hold promise for mitigating inflammation and enhancing systemic health through microbiome modulation.

## 1. Introduction

The human gut microbiome is increasingly recognized as a dynamic system that exerts far-reaching influences on host health. This vast community, comprising bacteria, archaea, and viruses, has co-evolved with its host to perform essential metabolic, immunological, and neurological functions [1,2]. Beyond simply aiding digestion, the microbiome facilitates energy harvesting from otherwise indigestible carbohydrates, synthesizes vitamins, metabolizes bile acids, and interacts with the host’s immune and endocrine systems [3]. A cornerstone of this regulation is the gut–brain axis (GBA), a bidirectional communication network linking the gastrointestinal tract with the central nervous system via endocrine, immune, autonomic, and neural pathways [3,4,5,6,7,8,9]. This complex network is crucial for regulating gastrointestinal functions, such as motility, secretion, and nutrient absorption; it also exerts a profound influence on higher brain functions, including mood, cognition, stress responses, and behavior [4]. The GBA allows microbial metabolites and immune signals to influence mood, cognition, and neurological health, while stress hormones and neural signals reciprocally shape gut physiology [6,7]. Disruptions to this balance, often through diet-induced dysbiosis, can propagate from the gut to the brain, contributing to systemic inflammation and neuropsychiatric disorders [10].

Within this complex ecosystem of gut microbiome, mostly in experimental mice, butyrate-producing bacteria (BPB)—primarily members of *Clostridium* clusters IV and XIVa—emerge as microbial sentinels due to their significant role in modulating gut and brain communication. These taxa, including *Faecalibacterium prausnitzii*, *Roseburia* spp., and *Eubacterium rectale*, specialize in fermenting dietary fibers and resistant starches into the short-chain fatty acid (SCFA) butyrate [11,12]. Butyrate plays a dual role: it is both a preferred energy substrate for colonocytes and a potent signaling molecule that enhances gut barrier integrity, regulates immune responses via histone deacetylase (HDAC) inhibition and G-protein-coupled receptor (GPCR) activation, and reduces neuroinflammation through GBA pathways [13,14,15,16]. In this sense, BPB acts as a keystone species: its metabolic activity stabilizes the gut environment and extends systemic benefits to host physiology. The clinical significance of BPB is reflected in its depletion across diverse chronic diseases. Lower abundances of BPB have been reported in inflammatory bowel disease (IBD), type 2 diabetes, obesity, metabolic syndrome, depression, Alzheimer’s disease, and Parkinson’s disease, with corresponding declines in butyrate production [8,13,14,17,18]. These recurring associations suggest that reduced butyrate bioavailability may serve as a unifying pathophysiological mechanism underlying disorders that span metabolic, inflammatory, and neurological domains. While we acknowledge the emerging evidence that minor SCFAs such as valeric acid contribute to neuroinflammatory regulation and epigenetic signaling within the gut–brain axis [19], the focal point of this review is butyrate, not as an exclusive indicator, but as a major contributor in the metabolic pathway regulating gut–brain axis signaling.

This paper explores how dietary inputs, ranging from macronutrients (carbohydrates, proteins, fats) to micronutrients and food additives, selectively shape BPB abundance and functionality. By integrating broad dietary mechanisms with specific insights into butyrate metabolism, this review situates BPB as a keystone taxa whose presence or depletion influences gut ecology, immune modulation, and gut–brain communication—ultimately, highlighting how nutrition can either preserve or undermine the role of BPB, with implications for health outcomes across multiple organ systems.

## 2. Materials and Methods

**Figure 1:**
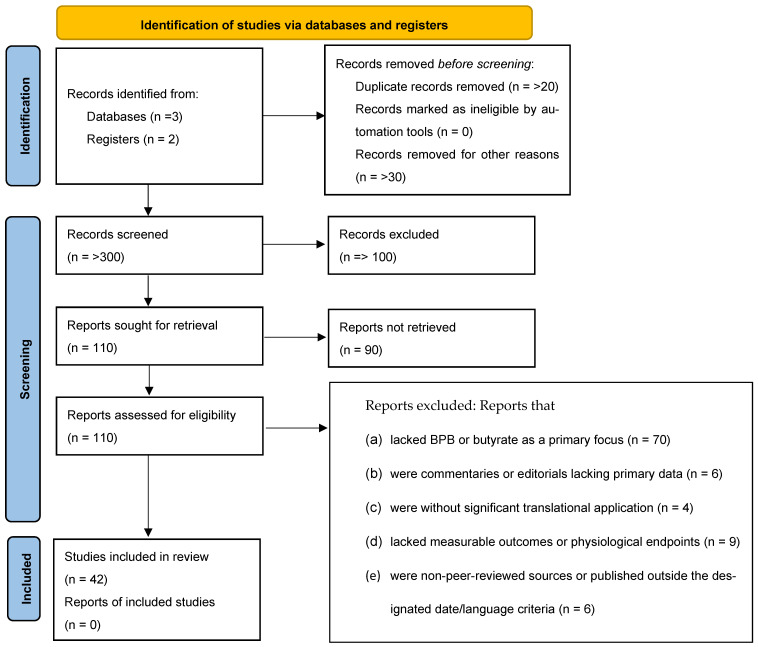
Flow diagram for thiis systematic review, which included searches of databases and registers only.

This review article was prepared in compliance with the PRISMA guidelines, which are published on the *IJMS* website (see the Supplementary Materials for details). A Flow diagram for thiis systematic review including the searches of databases and registers are shown in Figure 1.

### 2.1. Source of Data

Sources of evidence were considered if they met the following criteria:

- Population/Participants: Human-based studies (in vivo, clinical, or population level) examining the gut microbiome with a focus on butyrate-producing bacteria (BPB). Mechanistic animals or in vitro studies were included only when findings were directly linked to human health, as BPB functions are highly conserved and translational across models.
- Concept: Studies explicitly evaluate butyrate production, BPB abundance, or the functional effects of butyrate on host physiology (e.g., gut barrier integrity, inflammation, immune modulation, or gut–brain axis signaling).
- Context: Research was focused on dietary, microbial, or therapeutic influences on BPB prevalence and butyrate metabolism. This reflects the central role of diet, environment, and medical interventions (e.g., antibiotics) in modulating BPB prevalence.
- Study Design: Primary research article, including experimental, observational, and clinical studies. These were included to cover both mechanistic detail and population-level associations.
- Publication Criteria: Peer-reviewed, published in English, between 2013 and 2025.

Exclusion criteria were applied to eliminate studies that:

(a) Did not address BPB or butyrate as a primary focus (e.g., general microbiome surveys without relevant outcomes or discussion)
(b) Were reviews, commentaries, or editorials lacking new/current primary data.
(c) Focused exclusively on non-human microbiomes without significant translational application.
(d) Lacked measurable outcomes related to butyrate, microbial composition, or host physiological endpoints.
(e) Were non-peer-reviewed sources (e.g., theses, conference abstracts, gray literature) or published outside the designated date/language criteria, unless meeting the exception criteria below.

Exception Criteria:

While the primary eligibility window is 2013–2025, select pre-2013 were retained if they met at least one of the following criteria:

1. Foundational Mechanistic Discovery—First demonstration linking butyrate/BPB to epithelial barrier integrity, immune function, or host signaling pathways.
2. Seminal Conceptual Framework—Highly cited, field-defining works establishing the role of SCFAs/BPB in gut or systemic health, providing frameworks still relied on in the current literature.
3. Methodological Foundation—Papers introducing key analytical methods or defining BPB classifications that underpin subsequent research.

Pre-2013 Included Exceptions:

- Louis & Flint (2009) [11]—Criterion 1 and 2: Landmark paper on BPB taxonomy, metabolic ecology, and butyrate pathways; provides the mechanistic and ecological foundational evidence for nearly all the subsequent BPB literature.

These exceptions are flagged in the data charting table with “Exception = YES” and annotated with a rational category (1–3). Their inclusions ensure that critical mechanistic and conceptual frameworks are conserved—without broadening search criteria and risking inaccuracies or the non-current literature.

### 2.2. Database Search Strategy for Literature Review

Primary Databases and Tools: The following databases were the primary sources for the academic and clinical literature.

#### 2.2.1. PubMed/PubMed Central (PMC)

PubMed was the primary resource for the biomedical and clinical literature, utilizing its advanced search capabilities and MeSH (Medical Subject Headings) terms for precision.

• Strategy: Broad Foundational Search on BPB and Inflammation
○ Objective: To capture a wide range of articles linking gut microbiota, butyrate, and inflammation.

Search String:

((“Gastrointestinal Microbiome”[Mesh]) OR “gut microbiota”[tiab] OR “gut microbiome”[tiab]) AND ((“Butyrates”[Mesh]) OR “butyrate”[tiab] OR “butyrate-producing bacteria”[tiab]) AND ((“Inflammation”[Mesh]) OR “inflammation”[tiab] OR “Inflammatory Bowel Diseases”[Mesh])

○ Rationale: This search combines official MeSH terms with common keywords in the title/abstract ([tiab]) to create a comprehensive query that is both sensitive and specific.
• Strategy: Gut–Brain Axis and Neurological/Psychiatric Links
○ Objective: To find the literature connecting microbial butyrate production to neuroinflammation and mood disorders.

Search String:

((“Brain-Gut Axis”[Mesh]) OR “gut-brain axis”[tiab]) AND (“butyrate”[tiab] OR “short-chain fatty acids”[tiab]) AND ((“Neuroinflammatory Diseases”[Mesh]) OR “neuroinflammation”[tiab] OR “depression”[tiab] OR “anxiety”[tiab])

○ Rationale: This query targets explicitly the intersection of the GBA, microbial metabolites, and key neurological and psychiatric outcomes.
• Strategy: Therapeutic Interventions (Probiotics and Prebiotics)
○ Objective: To identify clinical trials and reviews on the use of probiotics and prebiotics to modulate butyrate and treat related diseases.

Search String: 

((“Probiotics”[Mesh]) OR “probiotics”[tiab] OR (“Prebiotics”[Mesh]) OR “prebiotics”[tiab]) AND (“butyrate”[tiab]) AND ((“Inflammatory Bowel Diseases”[Mesh]) OR (“Metabolic Syndrome”[Mesh]) OR “obesity”[tiab]) AND (Clinical Trial[ptyp])

○ Rationale: This search is filtered by publication type ([ptyp]) to “Clinical Trial” to focus on human intervention studies, which are crucial for assessing therapeutic potential.

#### 2.2.2. Scopus

Scopus was used for its broad index of high-impact and peer-reviewed literature.

• Strategy: Identification of Butyrate-Producing Bacteria (BPB)
○ Objective: To find the primary literature and reviews identifying the key bacterial producers of butyrate.

Search String: 

TITLE-ABS-KEY((“butyrate-producing bacteria” OR butyrogenic) AND (“faecalibacterium” OR “roseburia” OR “eubacterium” OR “clostridium cluster IV” OR “clostridium cluster XIVa”))

○ Rationale: This search uses the TITLE-ABS-KEY field to look for terms in the title, abstract, and keywords, and combines general terms with the names of known key BPB genera and clusters.
• Strategy: Butyrate’s Effect on Goblet Cells and the Mucus Barrier
○ Objective: To investigate the specific interaction between butyrate and the intestinal mucosal barrier.

Search String:

TITLE-ABS-KEY((butyrate) AND (“goblet cell*” OR mucin OR MUC2 OR “gut barrier”))

○ Rationale: The asterisk (*) is used as a wildcard to capture variations of “cell” (e.g., “cells”). This query hones in on the molecular and cellular components of the gut barrier.

### 2.3. Supplementary Databases and Tools

These resources were used for broader discovery, accessing full-text articles, and finding the foundational literature.

Google Scholar: Google Scholar’s strength is its broad indexing and citation tracking features. Searches were more conversational and phrase-based.

• Strategy: Broad Topic Discovery
○ Objective: To identify highly cited, seminal review articles that define the key concepts.
○ Search String: “review” “gut microbiota” “butyrate” “immune system regulation”
• Strategy: Citation Chaining (Non-String Method)
○ Objective: To trace the academic conversation forward from a known, important paper.
○ Process:
  1. Locate a foundational paper (e.g., Furusawa et al., 2013 [20], *Nature*, on butyrate and Tregs).
  2. Click the “Cited by” link on the search result.
  3. Filter the resulting list of ~1000+ papers by keyword (e.g., adding “microglia” to the search within citing articles) or sort by date to find the most recent research that builds upon the foundational findings.
○ Rationale: This is a powerful method for understanding the evolution of a research topic and ensuring the inclusion of the most current, relevant studies.

Elsevier (ScienceDirect): This database was used primarily to access full-text articles from key journals published by Elsevier.

• Strategy: Journal-Specific Advanced Search
○ Objective: To find relevant articles within top-tier journals.
○ Search Fields:
  ▪ “Terms”: butyrate AND “gut-brain axis.”
  ▪ “In journal”: *Gastroenterology* OR *Cell Host & Microbe*.
  ▪ “Date”: 2015–2025.
○ Rationale: Targeting high-impact journals helps prioritize the literature that has undergone rigorous peer review and is likely to be influential in the field.

## 3. Results and Discussion

### 3.1. Butyrate-Producing Bacteria (BPB) and the Multifaceted Roles of Butyrate

Butyrate, a major short-chain fatty acid (SCFA) produced by microbial fermentation of dietary fiber, exerts multidimensional benefits on host health. Within the colon, butyrate serves as the preferred energy substrate for colonocytes, fueling epithelial renewal and sustaining gut barrier integrity [13]. Adequate butyrate availability strengthens tight junction proteins, promotes mucin production through goblet cell stimulation, and induces the secretion of interleukin-18, which supports epithelial repair and defense [3]. Conversely, reduced butyrate levels impair these protective functions, predisposing the gut epithelium to increased permeability, commonly termed “leaky gut”, and heightening susceptibility to inflammation and infection [21,22].

Beyond its local metabolic role, butyrate functions as a signaling molecule that shapes immune homeostasis. Through histone deacetylase (HDAC) inhibition, butyrate promotes regulatory T-cell differentiation, dampens pro-inflammatory cytokine expression, and shifts immune responses toward tolerance [12]. In parallel, butyrate engages G-protein-coupled receptors (e.g., GPR41, GPR43) to modulate inflammatory signaling, further reinforcing its role as an anti-inflammatory metabolite [14,23]. This dual mechanism—epigenetic regulation and receptor-mediated signaling—positions butyrate as a key mediator of systemic immune balance. Butyrate’s influence extends beyond the gut through the gut–brain axis (GBA). Circulating SCFAs, particularly butyrate, can cross the blood–brain barrier or act peripherally on vagal afferents to regulate central nervous system activity [5]. Butyrate reduces neuroinflammation by modulating microglial activation, enhances blood–brain barrier integrity, and indirectly regulates neurotransmitter synthesis, including serotonin and gamma-aminobutyric acid (GABA) [6,24]. These interactions underscore the critical role of microbial metabolites in linking dietary inputs to cognitive and emotional states.

Clinically, butyrate deficiency has been associated with a broad spectrum of chronic disorders. Depletion of butyrate-producing bacteria is consistently observed in inflammatory bowel disease, type 2 diabetes, obesity, and metabolic syndrome, conditions characterized by impaired barrier function, systemic low-grade inflammation, and metabolic endotoxemia [10,17,25]. Neurological and psychiatric disorders, including depression, Alzheimer’s disease, and Parkinson’s disease, similarly demonstrate reductions in butyrate-producing taxa such as *Faecalibacterium* and *Coprococcus*, often accompanied by enrichment of pro-inflammatory microbes like *Eggerthella* [8]. This recurring pattern across metabolic, inflammatory, and neurological conditions suggests that butyrate functions as a central metabolic safeguard, and its loss may represent a common mechanistic thread linking disparate disease states.

There is substantial data to support a bidirectional relationship between stress and gut microbiota, particularly the BPB population. Chronic stress is associated with alterations in gut microbial composition, including reductions in SCFA-producing taxa, and HPA-axis activation can in turn modify intestinal barrier function and microbial ecology [26,27]. Emerging evidence suggests that microbiota-derived SCFAs, including butyrate, can modulate neuroendocrine stress responses by attenuating cortisol release and influencing neuroimmune signaling within the brain [28]. This reciprocal communication involving neural, endocrine, and immune pathways supports a bidirectional gut–brain axis in which stress can affect microbial community structure and microbial metabolites can shape host stress physiology [29].

Taken together, butyrate exemplifies how microbial metabolites can simultaneously maintain intestinal homeostasis, regulate immune responses, and shape brain function. Its systemic significance elevates butyrate-producing bacteria to the status of keystone species: their survival and activity are not only vital for gut ecology but also for preserving host health across multiple organ systems.

Butyrate-producing bacteria (BPB) constitute a functionally crucial, albeit phylogenetically diverse, group within the human gut microbiota [12,30]. They are predominantly Gram-positive, obligate anaerobic bacteria belonging to the Firmicutes phylum [14]. Taxonomically, the most significant butyrate producers are concentrated within two major clusters of the Clostridiales order: *Clostridium* cluster IV (also known as the *Clostridium leptum* group) and *Clostridium* cluster XIVa (the *Clostridium coccoides* group) [14]. Several key genera within these clusters are consistently identified as major contributors to butyrate production in the human colon and are presented in Table 1. Table 1 summarizes characteristics of prominent butyrate-producing bacterial genera found in the human gut microbiota based on the provided text. NGP = Next-Generation Probiotic. The table highlights key species, phylogenetic grouping (*Clostridium* Cluster), and notable metabolic or health-associated functions. Other butyrate-producing genera mentioned in the source text include *Butyrivibrio*, *Subdoligranulum*, and *Anaerobutyricum* [8,11,12,13,31,32,33,34].

The production of butyrate within the colon is a collective effort, distributed across a diverse array of bacterial taxa primarily within the Firmicutes phylum [30]. This functional redundancy, where multiple distinct species contribute to the same crucial metabolic output, likely confers resilience to the gut ecosystem. This distributed function contrasts with ecological roles that might depend solely on a single keystone species, highlighting the robustness built into this critical microbial function. Recent reviews highlight that BPB shapes microbial ecology and host responses beyond their role in SCFA generation, and that direct butyrate supplementation has shown mixed clinical efficacy, supporting a focus on microbial restoration strategies complemented by metabolite-targeted approaches [12,35,36].

### 3.2. Gut Microbial Environment as a Modulator of BPB Diversity and Activity

The intestinal environment is not a uniform habitat but a highly structured and dynamic ecosystem that dictates the viability and metabolic activity of butyrate-producing bacteria (BPB). Their ability to sustain host health depends on spatial organization, interactions with host-derived substrates such as mucus, cooperative metabolic exchanges with other microbes, and physicochemical gradients along the gastrointestinal tract. Together, these features create a finely tuned system in which BPB act as ecological stabilizers and metabolic hubs. Table 2 summarizes the diverse physiological functions of butyrate across multiple host domains. It highlights the dual role of butyrate as both an energy substrate and signaling molecule, its capacity to regulate barrier function and immunity, and its systemic influence on metabolism and the gut–brain axis. The table also links butyrate’s activity to broad disease protection across metabolic, inflammatory, and neurological conditions.

The mucus layer secreted by goblet cells provides one of the most critical habitats for BPB. This layer of the intestinal barrier is composed primarily of the mucin glycoprotein MUC2, which organizes into a dense inner layer and a looser outer layer [37,39]. The inner mucus layer is mainly sterile and shields epithelial cells from direct microbial contact, while the outer layer supports the growth of commensals, including BPB [38]. BPB not only occupies this niche but also directly influences its properties. Butyrate stimulates goblet cells to enhance mucin secretion and upregulates *MUC2* gene expression, thereby strengthening the protective barrier [37]. Moreover, butyrate synergizes with inflammatory cues such as interleukin-1β to promote the production of antimicrobial peptides, including cathelicidin LL-37, within the mucus layer [40,41]. This dual effect—reinforcing the mucus barrier and enriching its antimicrobial function—illustrates how BPB shapes host defenses at the mucosal surface. Disruption of this system has direct pathological consequences. In inflammatory bowel disease (IBD), goblet cell dysfunction and reduced mucin thickness are associated with diminished butyrate production, leading to impaired barrier integrity and heightened microbial translocation [39]. Conversely, restoration of BPB abundance through dietary fiber intake or prebiotics has been shown to normalize mucus dynamics, highlighting the therapeutic importance of the BPB–goblet cell axis.

BPB rarely acts in isolation but instead participates in complex microbial networks characterized by metabolic cross-feeding. Primary degraders such as *Bifidobacterium* spp. excel at hydrolyzing complex dietary fibers into acetate and lactate, which are then utilized by BPB as substrates for butyrate synthesis [12]. This interaction relies heavily on the butyryl-CoA/acetate CoA-transferase pathway, the dominant route of butyrate formation in the human colon [11]. Key BPB taxa, including *Faecalibacterium prausnitzii*, *Roseburia* spp., and *Eubacterium rectale*, act as secondary fermenters that convert acetate and lactate into butyrate, creating a metabolic loop that links dietary fiber availability to butyrate output [42]. Such cooperative interactions enhance metabolic efficiency and foster ecological stability. Even if one BPB species declines, others can compensate, maintaining relatively stable butyrate levels [30]. This redundancy underscores the resilience of the butyrate-production network. Perturbations in cross-feeding relationships, however, destabilize this system. Antibiotic use, for instance, disproportionately reduces *Bifidobacterium* alongside BPB such as *Faecalibacterium* and *Roseburia*, resulting in disrupted acetate supply and sharp declines in butyrate production [43]. Likewise, diets low in fermentable carbohydrates limit the substrates available for primary fermenters, weakening the entire network and reducing SCFA yield [44].

Beyond mucus and microbial networks, the spatial heterogeneity of the colon imposes powerful constraints on BPB activity. The proximal colon, where dietary fibers are most abundant, provides an acidic environment optimal for carbohydrate fermentation and butyrate production [13]. In contrast, the distal colon is nutrient-depleted and increasingly dominated by proteolytic fermentation, which yields potentially harmful metabolites such as ammonia, hydrogen sulfide, and phenolic compounds. These byproducts compromise epithelial integrity and create an environment less favorable for BPB survival. pH gradients are significant: mildly acidic conditions in the proximal colon enhance BPB activity, whereas alkaline shifts in the distal colon reduce butyrate output [13]. This gradient reflects the progressive depletion of fermentable substrates, making dietary fiber availability critical for sustaining butyrate production throughout the colon. Table 3 summarizes key ecological and host-derived factors that determine the abundance and activity of butyrate-producing bacteria. It highlights the importance of mucus niches, microbial cross-feeding, luminal gradients, and community redundancy in sustaining stable butyrate production.

The gut environment is therefore a modulator of BPB activity at multiple levels. Goblet cells establish physical and biochemical niches, cross-feeding ensures metabolic efficiency and redundancy, and luminal gradients define spatial zones of butyrate production. Disturbances in any of these features, whether through dietary changes, antibiotics, or disease, compromise butyrate availability and destabilize host–microbe interactions.

### 3.3. Dietary Patterns: Fiber vs. Western Diet and Their Impact on BPB

Diet is the dominant environmental factor shaping the composition, functionality, and resilience of the gut microbiome. While genetic and geographic factors exert measurable influences, the daily intake of macronutrients, micronutrients, and additives exerts the most immediate and sustained pressure on microbial communities, particularly butyrate-producing bacteria (BPB). The dietary landscape can therefore serve as either a scaffold for supporting BPB-mediated host health or a destabilizing force that depletes BPB and diminishes butyrate availability.

Dietary fiber represents the most important substrate for BPB, providing the fermentable carbohydrates necessary for butyrate synthesis. Resistant starches, inulin, and other soluble fibers escape enzymatic digestion in the small intestine and reach the colon, where BPB metabolize them via fermentation pathways to produce butyrate as the major product [13]. *F. prausnitzii*, *R. intestinalis*, and *E. rectale* are particularly enriched in individuals consuming high-fiber diets and act as central mediators of fiber-to-butyrate conversion [12]. Fiber not only sustains BPB but also enriches cooperative networks. *Bifidobacteria* and other primary degraders generate acetate and lactate, which feed secondary BPB through cross-feeding loops [11]. This interdependence underscores why fiber deprivation results in a rapid decline in both BPB abundance and overall butyrate production. Clinical studies consistently show that high-fiber diets are associated with reduced risk of inflammatory bowel disease, obesity, and type 2 diabetes, mediated in part through restoration of SCFA balance and reinforcement of epithelial barrier integrity [17]. Spatially, fiber fermentation occurs predominantly in the cecum and proximal colon, producing local pH conditions that favor BPB colonization and suppress pathogenic taxa [13]. By contrast, low fiber intake shifts fermentation distally, where proteolytic metabolism dominates, leading to increased production of toxic byproducts such as ammonia, hydrogen sulfide, and phenols that can erode barrier function [44]. Thus, fiber intake not only determines BPB abundance but also the spatial distribution of butyrate synthesis along the colon.

In contrast to fiber-enriched diets, Western-style dietary patterns characterized by low fiber and excessive fat and protein intake consistently erode BPB populations. High-fat diets increase the relative abundance of bile-tolerant microbes while reducing obligate anaerobes such as BPB [44]. This shift is driven by bile acid metabolism: elevated bile salts act as selective agents against fiber-fermenting anaerobes, while favoring pathobionts capable of bile acid tolerance [44]. Saturated fats are particularly deleterious, as they not only diminish BPB but also disrupt epithelial tight junctions, increasing gut permeability and fueling systemic endotoxemia [2]. Excess dietary protein compounds this effect. When fiber availability is low, colonic microbes increasingly ferment protein, producing metabolites such as ammonia, hydrogen sulfide, and branched-chain fatty acids. These compounds impair colonocyte metabolism, weaken tight junctions, and can directly suppress BPB growth. Elevated hydrogen sulfide, for instance, inhibits butyrate oxidation in colonocytes, further compromising epithelial health. This not only reduces butyrate availability but also generates a toxic environment that perpetuates inflammation and promotes disease. Such dietary imbalances are strongly linked to metabolic syndrome and type 2 diabetes. Reduced BPB populations and altered SCFA profiles have been repeatedly observed in obese and diabetic patients, accompanied by increased gut permeability and chronic low-grade inflammation [10,13]. The combination of low fiber and high fat/protein, therefore, represents a “double hit” against BPB, simultaneously starving them of substrates and enriching competitors that thrive under altered bile acid and proteolytic conditions.

The Western diet (WD), defined by high intake of refined carbohydrates, saturated fats, and processed foods, exerts cumulative and synergistic effects on the gut microbiome. Reduced microbial diversity, loss of keystone taxa such as BPB, and enrichment of pathobionts are hallmarks of “WD-induced dysbiosis” [44]. The decline in butyrate output is of particular concern, as it underlies many of the systemic effects associated with WD, including impaired barrier function, metabolic endotoxemia, and chronic inflammation. Population-level data underscore this burden. Obesity and metabolic syndrome, both tightly linked to WD, are associated with a reproducible reduction in *Faecalibacterium* and *Roseburia* abundance [13]. This depletion translates into diminished butyrate production, which, in turn, compromises epithelial repair and propagates inflammatory signaling. The resulting cycle of dysbiosis, leaky gut, and systemic inflammation contributes not only to metabolic disease but also to the development of neuropsychiatric disorders via GBA dysregulation [8]. The Western diet is also heavy in food additives and preservatives consumed through processed and preserved food items. Some of the impacts of WD on BPB could be attributed to these additives. For example, *Facalibacterium* is susceptible to polysorbate-80, while cinnamaldehyde promotes the growth of this taxon in the human gut [45]. Sorbitol, xylitol, and sodium benzoate are other examples of food additives that alter the abundance of different BPB in the gut [46,47,48].

The evidence converges on a central theme: diet exerts a bidirectional influence on BPB and butyrate production. Fiber-rich diets create ecological niches and metabolic networks that sustain BPB, reinforcing gut integrity, immune regulation, and neuroimmune signaling. By contrast, low-fiber, high-fat/protein diets and widespread additive exposure erode BPB populations, reduce butyrate availability, and propagate systemic disease risk. The dietary modulation of BPB thus acts as a keystone determinant of host health.

### 3.4. Influence of Antibiotics on BPB Abundance and Activity

Antibiotics, while crucial for treating bacterial infections, are potent disruptors of the gut microbiota. Their use, particularly broad-spectrum antibiotics, can lead to significant short-term and sometimes long-term consequences such as marked reduction in microbial species diversity and abundance, alterations in the metabolic activity like SCFA production, including butyrate, and the selection pressure favoring the proliferation of antibiotic-resistant bacteria [43]. Antibiotic treatment significantly depletes key populations of butyrate-producing bacteria, belonging to the *Ruminococcaceae* and *Lachnospiraceae* families, including *Faecalibacterium* and *Roseburia* [43]. While the microbiota may partially recover following antibiotic cessation, recovery can be slow, and some beneficial species may be permanently lost or remain at significantly reduced levels, leading to persistent dysbiosis. This long-term dysbiosis is epidemiologically linked to an elevated risk of developing chronic conditions later in life, including IBD, obesity, Type 2 diabetes, asthma, and allergies. Mechanistically, antibiotic-induced dysbiosis can promote inflammation by damaging the intestinal barrier, allowing the translocation of bacterial products like lipopolysaccharides (LPS), and by increasing the relative abundance of pro-inflammatory pathobionts while reducing the abundance of beneficial, immunomodulatory bacteria [49]. While essential for combating infections, antibiotics often lack specificity and can indiscriminately eliminate both pathogenic and beneficial commensal bacteria. Together, this significantly impacts BPB populations and butyrate production. Crucially, many antibiotics, particularly broad-spectrum agents or those targeting anaerobes (like clindamycin or metronidazole), cause a marked depletion of key butyrate-producing taxa within the Firmicutes phylum, including members of the *Ruminococcaceae* and *Lachnospiraceae* families such as *Faecalibacterium* and *Roseburia*. This loss of BPB directly translates to reduced butyrate production in the colon [43,50]. Strategies to mitigate the negative impact of antibiotics on BPB and butyrate production are currently being researched and are considered a relatively new area of research. These may include the use of more targeted antibiotics when possible, co-administration of probiotics (although evidence for preventing dysbiosis is still debated), or post-antibiotic interventions such as prebiotics or fecal microbiota transplant (FMT) to aid microbiota recovery.

Considering the established roles of diet and antibiotics in shaping the gut microbiota, as well as the links between dysbiosis and chronic inflammatory and metabolic conditions, the confluence of factors prevalent in the US lifestyle presents a compelling area for investigation. The widespread consumption of Western diets rich in protein and fats, which may also contain additives restricted in other regions due to safety concerns, combined with the significant use of antibiotics, may promote a state of chronic gut dysbiosis within the population. This microbially mediated disruption could contribute to the high burden of obesity, metabolic syndrome, and other inflammatory conditions observed, thereby partially explaining the nation’s challenging health expenditure and outcome statistics [43].

### 3.5. Scoping for the Future

The recognition of butyrate-producing bacteria (BPB) as keystone taxa has profound implications for therapeutic strategies. Their capacity to safeguard epithelial integrity, regulate immune tolerance, and modulate gut–brain communication makes them a central target for dietary and clinical interventions. As evidence accumulates linking BPB depletion to chronic inflammatory, metabolic, and neurological disorders, strategies to restore their abundance and function are becoming a critical focus in microbiome research and translational medicine.

The most straightforward approach to restoring BPB function is through diet. Supplementation with fermentable fibers and resistant starches provides substrates that selectively enrich BPB populations. Controlled feeding studies demonstrate that diets enriched in resistant starch significantly increase the abundance of *F. prausnitzii* and *Roseburia* spp., with corresponding increases in butyrate output and improvements in barrier integrity [12,13]. Prebiotics such as inulin, fructooligosaccharides (FOSs), and galactooligosaccharides (GOSs) have been shown to stimulate cross-feeding networks between bifidobacteria and BPB, enhancing acetate-to-butyrate conversion [11]. Significantly, the benefits extend beyond local gut effects: increased butyrate levels are associated with systemic improvements in glucose metabolism, lipid profiles, and inflammatory markers [17]. The concept of “fiber thresholds” is emerging, emphasizing that a minimum level of daily fiber intake is necessary to sustain butyrate synthesis across the colon. Falling below this threshold leads to the collapse of mucus-enriched niches, the erosion of protective barriers, and the expansion of proteolytic fermentation [44]. Though there is enough data to support the dose–response relationship between dietary fiber and BPB maintenance, there is no existing mathematical model to predict a fiber-threshold to sustain BPB across different populations. Identifying optimal fiber thresholds for individual populations is, therefore, a critical next step in clinical translation.

Traditional probiotic formulations have had limited success in increasing BPB abundance, as most commercially available strains are not robust butyrate producers. However, symbiotic strategies—combining prebiotics with probiotic strains that synergize with BPB—show greater promise. For instance, supplementation with *B. adolescentis* alongside inulin can increase butyrate levels by enhancing cross-feeding networks [42]. More innovative approaches focus on next-generation probiotics, such as live biotherapeutic products containing *F. prausnitzii* or *R. intestinalis*. Early trials have demonstrated feasibility, though challenges remain in formulating oxygen-sensitive strains for clinical use. Encapsulation technologies and anaerobic delivery systems may overcome these barriers, enabling targeted restoration of BPB in the colon [43].

Fecal microbiota transplantation (FMT) has emerged as a powerful tool for restoring microbial ecosystems in patients with recurrent *Clostridioides difficile* infection. It is now being investigated for broader indications, including inflammatory bowel disease and metabolic syndrome. Studies reveal that successful engraftment is often characterized by the restoration of BPB populations, with post-transplant increases in *Faecalibacterium* and *Roseburia* correlating with clinical improvements [10]. However, outcomes are variable, and donor–recipient compatibility strongly influences success. Individuals with baseline microbiomes that are highly depleted of BPB may require repeated transplants or adjunctive dietary interventions to sustain engraftment. Future developments in FMT are likely to shift toward defined microbial consortia, designed to include stable BPB strains in combination with supporting taxa to maximize ecological resilience.

Inter-individual variability in microbiome composition, genetic background, and diet strongly influences responses to interventions aimed at restoring BPB. For example, sensitivity to common emulsifiers such as carboxymethylcellulose varies between individuals, with some showing profound shifts in microbial composition and others demonstrating resilience. Baseline microbiome profiling may predict these sensitivities, enabling personalized dietary recommendations that limit exposure to harmful additives while emphasizing BPB-supporting nutrients. This customized approach extends to prebiotic supplementation. Not all individuals respond equally to the same fiber or prebiotic, as efficacy depends on the presence of primary degraders and the strength of cross-feeding networks. Incorporating microbiome sequencing and metabolic profiling into dietary planning could allow clinicians to prescribe tailored interventions that selectively enrich BPB and maximize butyrate production.

Restoring BPB abundance is not only about repairing gut ecology but also about addressing systemic disease risk. Evidence suggests that interventions enhancing butyrate production may reduce inflammation, improve insulin sensitivity, and mitigate neuroinflammatory processes implicated in depression and Alzheimer’s disease [8]. The translational challenge lies in identifying scalable, safe, and effective interventions that can be adapted across diverse populations and dietary environments.

To explore these options, future research could focus on the following:

1. Defining fiber thresholds necessary for sustained butyrate synthesis across different populations.
2. Developing stable formulations of next-generation probiotics containing BPB.
3. Integrating microbiome-informed diagnostics to tailor prebiotic, symbiotic, and dietary interventions.
4. Re-evaluating additive safety regulations to account for their impact on significant microbial species, rather than just host toxicity.

By centering BPB as a keystone organism, the field can move beyond correlational associations toward causal, mechanism-driven interventions that leverage diet and microbial therapeutics to sustain systemic health.

## 4. Conclusions

The human gut microbiome represents a complex and adaptive ecosystem, and within this system, butyrate-producing bacteria (BPB) function as keystone species. By converting dietary fibers into the short-chain fatty acid butyrate, these microbes act as metabolic sentinels that uphold barrier integrity, regulate immune balance, and influence neurological health through the gut–brain axis. Their activity exemplifies the principle that a small subset of microbial taxa can exert disproportionate influence on host physiology, stabilizing not only gut ecology but also systemic homeostasis. The evidence reviewed here demonstrates that BPB abundance and butyrate production are susceptible to dietary patterns. Fiber- and prebiotic-rich diets reinforce BPB niches and cross-feeding networks, producing ecological stability and systemic resilience.

In contrast, Western-style diets characterized by low fiber and high fat/protein intake erode BPB populations, shift microbial metabolism toward proteolysis, and generate toxic byproducts that weaken barrier function and promote systemic inflammation. Food additives further exacerbate this vulnerability by directly suppressing BPB or disrupting the mucus barrier. Together, these factors position diet as a double-edged sword—capable of nurturing microbial keystone species or accelerating their depletion with profound consequences for host health. The depletion of BPB and the decline of butyrate production emerge as recurring signatures across diverse chronic conditions, including inflammatory bowel disease, type 2 diabetes, obesity, metabolic syndrome, depression, Alzheimer’s disease, and Parkinson’s disease. These consistent patterns suggest that butyrate deficiency may represent a unifying mechanistic link among inflammatory, metabolic, and neurodegenerative disorders.

Looking ahead, strategies such as prebiotic supplementation, symbiotic formulations, next-generation probiotics, and fecal microbiota transplantation offer promising avenues for restoring BPB populations. Yet, inter-individual variability in microbiome composition underscores the need for personalized approaches. Advances in microbiome sequencing, metabolic profiling, and ecological modeling will enable clinicians to identify fiber thresholds, predict additive sensitivities, and design tailored dietary and microbial interventions. Regulatory frameworks must also evolve to incorporate microbial health when evaluating the safety of food additives and preservatives. The health of the host and the resilience of the microbiome are intimately intertwined through the keystone functions of butyrate-producing bacteria. By framing BPB as central ecological regulators and by recognizing diet as their primary determinant, this review highlights a path forward: harnessing nutritional strategies and microbial therapeutics to preserve butyrate synthesis as a cornerstone of systemic health. Overall, this review summarizes the potential use of dietary strategies that sustain BPB as a cornerstone in preventing chronic inflammation and neurodegenerative diseases.

## Figures and Tables

**Table 1:** Key butyrate-producing bacterial genera in the human gut and their characteristics.

Genus	Key Species Mentioned	Clostridium Cluster	Key Characteristics and Functions
***Faecalibacterium***	*F. prausnitzii*	IV	Highly abundant (often 5–15% + of total fecal bacteria); potent anti-inflammatory properties; biomarker of intestinal health; potential next-generation probiotic.
***Roseburia***	*R. intestinalis*, *R. hominis*, *R. inulinivorans*	XIVa	Significant butyrate producer via fermentation of dietary fibers and cross-feeding on acetate; *R. intestinalis* is noted for mucus adhesion.
***Eubacterium***	*E. rectale*, *E. hallii*	IV and XIVa	Abundant butyrate producers utilize dietary fibers, acetate, and lactate for butyrate synthesis.
***Anaerostipes***	*A. caccae*, *A. hadrus*	XIVa	Convert lactate and acetate into butyrate, contributing to cross-feeding interactions.
***Coprococcus***	*C. eutactus*	XIVa	Ferments carbohydrates and utilizes acetate/lactate for butyrate production; reduced levels have been linked to depression and neurological disorders.
***Clostridium***	*C. butyricum* (non-pathogenic probiotic species)	I	Includes non-pathogenic butyrate producers like *C. butyricum*, which has been utilized as a probiotic, particularly in Asia. (Note: Genus also contains well-known pathogens.)

**Table 2:** Physiological function/significance overview of butyrate.

Physiological Domain	Butyrate Function	Mechanism(s)	Key Outcomes	References
Gut Epithelium	Primary fuel for colonocytes	Oxidation of butyrate in epithelial cells	Maintains epithelial renewal and integrity	[3,13]
Barrier Function	Strengthens mucus and tight junctions	Goblet cell stimulation; MUC2 upregulation; IL-18 induction	Prevents “leaky gut”; limits microbial translocation	[37,38,39]
Immune Regulation	Anti-inflammatory signaling	HDAC inhibition; GPCR activation; Treg differentiation	Reduces pro-inflammatory cytokines; promotes immune tolerance	[12,14]
Metabolism	Energy balance and satiety regulation	SCFA-mediated GLP-1 and PYY release; cross-feeding with acetate/lactate	Improves insulin sensitivity; regulates appetite and glucose homeostasis	[10,17]
Gut–Brain Axis	Neuroimmune and neurochemical modulation	Microglial regulation; serotonin and GABA synthesis; BBB protection	Reduces neuroinflammation; influences cognition and mood	[5,6,8]
Disease Protection	Broad systemic defense	Sustains BPB populations and butyrate output across systems	Reduced risk of IBD, T2D, obesity, depression, AD, PD	[10,13,14]

**Table 3:** Ecological and host-derived factors as determinants for BPB availability.

Environmental Factor	Microbial/Host Interaction	Effect on Butyrate Production	References
Goblet Cells and Mucus	*MUC2* secretion provides a niche for BPB; butyrate upregulates mucin production	Enhances mucus barrier integrity; supports BPB colonization	[37,38,39]
Cross-Feeding	*Bifidobacterium* produces acetate/lactate, which BPB utilizes	Increases the efficiency of butyrate synthesis via the CoA-transferase pathway	[11,12]
Luminal pH Gradient	Proximal colon = acidic, fiber-rich; distal colon = proteolytic	Acidic pH favors butyrate production; proteolysis reduces SCFAs and increases toxic byproducts	[13]
Microbial Redundancy	Multiple taxa produce butyrate via overlapping pathways	Maintains stable butyrate output despite species fluctuations	[30]

## Data Availability

No new data were created or analyzed in this study. Data sharing is not applicable to this article.

## Supplementary Materials

The following supporting information can be downloaded at: https://www.mdpi.com/article/10.3390/ijms27031289/s1. Reference [51] is cited in the supplementary materials.

## Abbreviations

The following abbreviations are used in this manuscript:

BPB	Butyrate-producing bacteria
GBA	Gut–brain axis
SCFA	Short-chain fatty acid
HDAC	Histone deacetylase
GPCR	G-protein-coupled receptor
IBD	Inflammatory bowel disease
GABA	Gamma-aminobutyric acid
WD	Western diet
LPS	Lipopolysaccharide
FMT	Fecal microbiota transplant
